# Determining the quality control frequency of an MR‐linac using risk matrix (RM) analysis

**DOI:** 10.1002/acm2.13984

**Published:** 2023-04-24

**Authors:** Min Ma, Hui Yan, Minghui Li, Yuan Tian, Ke Zhang, Kuo Men, Jianrong Dai

**Affiliations:** ^1^ National Cancer Center/National Clinical Research Center for Cancer/Cancer Hospital, Chinese Academy of Medical Sciences and Peking Union Medical College Beijing China

**Keywords:** failure mode and effect analysis, frequency, quality control, risk matrix

## Abstract

**Purpose:**

Quality control (QC) is performed routinely through professional guidelines. However, the recommended QC frequency may not be optimal among different institutional settings. Here we propose a novel method for determining the optimal QC frequency using risk matrix (RM) analysis.

**Methods and materials:**

A newly installed Magnetic Resonance linac (MR‐linac) was chosen as the testing platform and six routine QC items were investigated. Failures of these QC items can adversely affect treatment outcome for the patient. Accordingly, each QC item with its assigned frequency forms a unique failure mode (FM). Using FM‐effect analysis (FMEA), the severity (S), occurrence (O), and detection (D) of each FM was obtained. Next, S and D based on RM was used to determine the appropriate QC frequency. Finally, the performance of new frequency for each QC item was evaluated using the metric E = O/D.

**Results:**

One new QC frequency was the same as the old frequency, two new QC frequencies were less than the old ones, and three new QC frequencies were higher than the old ones. For six QC items, E values at the new frequencies were not less than their values at the old frequencies. This indicates that the risk of machine failure is reduced at the new QC frequencies.

**Conclusions:**

The application of RM analysis provides a useful tool for determining the optimal frequencies for routine linac QC. This study demonstrated that linac QC can be performed in a way that maintains high performance of the treatment machine in a radiotherapy clinic.

## 1 INTRODUCTION

Routine quality control (QC) of a linac aims to evaluate the states of various machine parameters and to identify any potential equipment defects or malfunctions through specifically designed tests. With the introduction of advanced treatment technologies, the number of QC items has dramatically expanded in recent years, which requires medical physicists to invest more time and effort in QC. The QC of a linac is usually performed regularly based on guidelines recommended by professional societies such as AAPM TG142,^1^ AAPM TG148.^2^ However, this regular QC frequency may not be appropriate because the states of the linacs (age, type, manufacturer, the number of treated patient) can be quite varied. Consequently, there is a need to find the optimal QC frequencies for a given machine to maintain its performance while minimizing physicists’ efforts.

Risk management has recently been attracting an increased amount of attention in the radiotherapy community. Risk management involves the systematic application of management rules, methods, and practices to the tasks of analyzing, evaluating, controlling, and monitoring risk.^3^ Failure mode and effect analysis (FMEA) is one of the most commonly used tools to identify, assess, and prioritize the steps that may cause risks in the radiotherapy process. Risk management has been mainly applied in two areas of radiotherapy: (1) planning and delivery processes and (2) linac quality assurance (QA) and QC. Each area has its own unique characteristics and parameters, with specially designed tests and data analysis tools.

Several investigators have attempted to identify weaknesses in the treatment planning and delivery of radiotherapy using risk management tools. Huq et al.^4^ and Ford et al.^5^ first proposed carrying out FMEA for conventional external‐beam radiation therapy to understand the possible sources of errors. Later, they simplified the FMEA for intensity‐modulated radiation therapy (IMRT) to reduce the time‐consuming process involved.^6^ Broggi et al.^7^ and Jones et al.^8^ implemented FMEA to identify the high‐risk step in the design of a tomotherapy plan. Noel et al.^9^ performed FMEA to analyze the risks introduced in adaptive radiotherapy (ART). FMEA has also been used to improve quality management in stereotactic body radiotherapy (SBRT) and stereotactic radiosurgery (SRS),^10^, ^11^, ^12^, ^13^, ^14^ proton‐beam radiotherapy,^15^, ^16^ internal skin irradiation,^17^ intrapelvic irradiation,^18^ and intraoperative electron radiation therapy.^19^, ^20^ It has been shown that higher quality and better safety levels can be achieved if proactive steps and processes are taken well before deployment.^21^

Risk management tools have also been used to evaluate priorities of linac QC items. O'Daniel et al.^22^ investigated the FMEA approach in a quantitative analysis of linac QC to determine the frequency of daily QC items. Bonfantini et al.^23^ applied FMEA to optimize the protocols of linac QC to ensure patient safety and treatment quality. In 2016, the American Association of Physicists in Medicine (AAPM) issued the report of Task Group 100 (the TG‐100 report),^24^ which introduced the principles of FMEA and related risk management tools in detail.^25^ Recently, FMEA has become a useful tool for evaluating the prioritization of QA testing for linacs. Thus, FMEA has had many successful applications in ranking the QC items in the QA process. However, the frequency of linac QC has rarely been investigated quantitatively in current publications. Too high a frequency of linac QC requires more effort by medical physicists and causes an increased workload, so an optimal QC frequency is always sought to obtain the best efficiency in busy radiotherapy clinics.

Risk matrix (RM) analysis has been successfully applied in many engineering fields, and it is an ideal tool for addressing this issue. It employs a semiquantitative method to evaluate the likelihood and severity of each FM using a two‐dimensional matrix.^26^ Vilaragut et al.^27^ employed RM analysis to classify event sequences in several hospitals. Their process allowed for self‐evaluation and for the management of safety measures that were most appropriate for each institution's conditions. Zhang et al.^28^ used RM to visualize the risk of suicide within 5 years after a lung cancer diagnosis, which could be promising for preventing unnecessary suicides among lung cancer patients. Klüter et al.^29^ used RM to establish risk levels that reflected on the institution risk strategy for online adaptive radiotherapy using a hybrid MR‐linac. Their approach established risk reduction priorities in a systematic and relatively easy manner.

Linac QC is generally performed on a daily, weekly, monthly, or annual basis, according to guidelines recommended by professional societies such as the AAPM.^1^, ^2^ However, it has long been argued^1^, ^2^ whether the recommended linac QC items are validated and appropriate for routine clinical practice. In this study, we propose a novel method for determining the linac QC frequency by applying RM analysis. This paper is structured as follows: Section 2 introduces the details of the MR‐linac settings, the six QC items, patient information, and then describes the derivation of indices for the severity (S), occurrence (O), and detection (D), together with the principles of RM analysis. Section 3 reports the new QC frequencies and compares them to the old frequencies. Section 4 discusses the advantages and disadvantages of this method.

## 2 METHODS AND MATERIALS

### 2.1 Testing platform

The MR‐linac is a medical device that combines magnetic resonance imaging (MRI) and radiotherapy functions in a single machine. In May 2019, an Elekta Unity MR‐linac system was installed in our institute. This system includes a 7 MV flattening filter‐free beam linac (Elekta AB, Stockholm, Sweden) and a 1.5 T MRI scanner (Philips Medical Systems, Best, The Netherlands). The acceptance test and machine commissioning were completed in 4 months. Thereafter, 14 patients were treated on the Unity MR‐linac in clinical trials. We started to treat patients on a regular basis using this machine in June 2020. By the end of December 2021, about 86 patients had been treated on this machine.

Machine QA has been performed on this MR‐linac since June 2020, when the first patient treatment started. Six QC items are performed monthly to check the machine status. They are the gantry angle indicator (QC1), the coincidence between collimator and couch (QC2), the jaw‐position indicator (QC3), the treatment‐couch position indicator (QC4), the size of the radiation isocenter (QC5), and the MR‐to‐MV fit (QC6). Daily and annual checking of the machine have also been performed, but those results are not listed here.

In 2021, the Elekta MR‐linac consortium published a QA report for the Unity MR‐linac.^30^ This report contains QC guidelines recommended by the machine vendor. Table 1 lists the QC tolerances used in our institute and those recommended in the vendor report. The indices QC1–QC5 are the same as the corresponding ones used for a conventional linac. Because the on‐board imaging system for this machine is MRI instead of cone‐beam computed tomography, we can expect the MR image center and the portal image center of the Unity MR‐linac has not been changed. This QC item is different from that for a conventional linac and is checked in item QC6.

**TABLE 1:** The QC items used in our institute and recommended by vendor report.

	Tolerance		
Monthly QC items	Institution	Elekta	Modeled Error
1. Gantry angle indicator	0.2°	0.3°	0.2° changes in gantry angle
2. Coincidence collimator–couch	0.2°	0.2°	0.2°changes in gantry angle
3. Jaw position indicator	1.0 mm	1.0 mm	1.0 mm shift in jaws
4. Couch position precision	1.0 mm	1.0 mm	1.0 mm shift in AP direction
5. Radiation isocenter size	0.5 mm	0.5 mm	0.5 mm translational shift in three axes
6. MR to MV fit	1.0 mm	1.0 mm	1.0 mm translational shift in three axes

The modeled errors are the error introduced to mimic the severity of the model failure for linac QC.

AP, anterior‐posterior.

### 2.2 Patient information

We randomly selected ten IMRT patients with treatment sites in the head (two cases), thorax (three cases), abdomen (four cases), and pelvis (one case). They had been previously treated on the Unity MR‐linac (Elekta AB, Stockholm, Sweden) in our institute between June 2020 and December 2021. All treatment plans were generated using the graphics processing unit Monte Carlo dose (GPUMCD) algorithm in the Monaco treatment planning system (Version 5.4, Elekta AB, Stockholm, Sweden). We used the adapt‐to‐position adaptation method for the selected patients. The MLC segments are translated in adapt‐to‐position. Table 2 summarizes the characteristics of these patients.

**TABLE 2:** The characteristics of 10 patients selected in this study.

Patient No.	Age	Cancer	Prescribed dose (cGy)	Fractions
1	49	Colon Cancer	4400	11
2	56	Retroperitoneal lymph nodes	6000	25
3	39	Intracranial B‐cell lymphoma	3000	15
4	66	Lung cancer	5200	13
5	74	Breast cancer	4000	10
6	66	Nasopharyngeal Carcinoma	2000	10
7	51	Liver Cancer	4400	22
8	60	Endometrial malignant tumor	6300	13
9	85	Colon Cancer	5000	10
10	58	Breast cancer	5000	25

### 2.3 Calculating the S, O, and D index

We tested each QC item at six frequencies: daily (D1), weekly (D7), biweekly (D14), triweekly (D21), monthly (D30), and bimonthly (D60). A QC item tested at a given frequency, together with its assigned tolerance, forms a FM. Each FM can be represented in the form FM (QC_m_, D_n_), where m is the index of the QC test, and n is the interval days of the QC tests. We calculated the quantities of failures for S from the dose differences, for O from the failure frequency, and for D from the probability of failure going undetected. These values were then assigned ranking numbers according to the criteria recommended by the AAPM TG‐100 report,^24^ as shown in Table 3.

**TABLE 3:** Threshold values specified for S, O, and D

	Severity (S)			
Rank	Change in % PTV	Change in max spinal cord dose	Occurrence (O)	Detectability (D)
1	≤1%	≤Δ45cGy	≤0.01%	≤0.01%
2	≤2%	≤Δ90cGy	≤0.02%*	≤0.2%
3	≤3%	≤Δ135cGy	≤0.05%	≤0.5%
4	≤4%	≤Δ180cGy	≤0.1%	≤1%
5	≤5%	≤Δ225cGy	≤0.2%	≤2%
6	≤10%	≤Δ450cGy	≤0.5%	≤5%
7	≤15%	≤Δ675cGy	≤1%	≤10%
8	≤20%	≤Δ900cGy	≤2%	≤15%
9	≤50%	≤Δ22.5Gy	≤5%	≤20%
10	≥50%	≥Δ22.5Gy	≥5%	≥20%

* Rank 2 is for errors that were never detected. The quantity of failure for the entire period (ten more years) was assumed to be less than 0.02 instead of 0.01 if the QC item was within tolerance. [Correction added on May 5, 2023, after first online publication: table 3 is updated.]

The quantity of failure for S was obtained by introducing QC related errors in the original plan and their effects on the dose were evaluated. The QC related errors are shown in the fourth column of Table 1. The magnitude of each modelled error was the level of tolerance in a copy of the plan, which was named as “the modified plan.” The modeled error of all these FMs was carried out in a same way in each plan fraction. The variation in the percentage of PTV covered by the prescription dose and the variation in the maximum dose to the spinal cord between the modified and original plans were obtained. The mean dose variations between the modified and original plans over 10 patients were used as the quantity of failure for S. These were adapted plans for the same patient but taken from a different day. All plans were normalized to cover at least 95% of the planning target volume (PTV) with the prescribed dose. Table 3 shows the characteristic of 10 IMRT patients treated on Unity MR‐linac.

The quantity of failure for O was obtained from the frequency of QC records for which the measurements exceeded the tolerance in the past 1.5 years (18*12*20 = 360d, 20 days within a month). The O value is the ratio of the time exceeding the tolerance to the total time. The quantity of failure for D was calculated based on the QC and service records. If a failure was not found in the current QC test but was found before the next QC test, we assumed the duration of the failure to be half of the time between the end of the current QC and the time the failure was found. The ratio of the total duration of failures to the total duration of QC tests is D.

### 2.4 Determining the QC frequency

**FIGURE 1:**
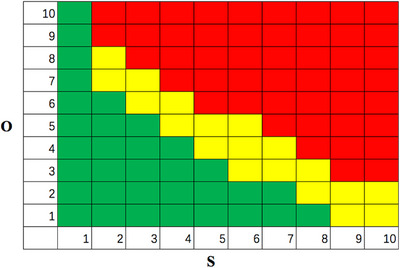
The reference RM recommended in the AIAG and VDA FMEA handbook.

The RM is a two‐dimensional look‐up table for assessing the risk of a given FM from the ranks of the indices O and S. It has long been studied in critical applications such as air traffic control. According to the Automotive Industry Action Group (AIAG) and the Verband der Automobilindustrie (VDA) FMEA handbook (2019),^31^ the reference RM is well established, as shown in Figure 1. In order to use the same S and O index as was recommended by AAPM TG‐100 report,^24^ the RM adopts a 10×10 risk table with severity along the X‐axis and occurrence along the Y‐axis. The 100 combinations of S and O values were classified into 3 categories: high risk (red), medium risk (yellow), and low risk (green).^32^ The criteria for risk categories were as followa.^32^ When the RM believes that S =1 or O =1, the degress of risk is considered to be low; S = 2‐5, O + S = 9‐10 corresponds to medium risk, 11‐15 corresponds to high risk; S = 6‐10, O +S = 9‐11 corresponds to medium risk; 12‐20 corresponds to high risk. According to the AAPM TG100 report, high S values should be considered separately; therefore, we consider O = 1, S = 9 and S = 10 as medium risk. Cells in the red zone are unacceptable, and further action is required. Cells in the yellow zone may be accepted under defined mitigation conditions. Cells in the green zone may be accepted without further action.^26^, ^33^, ^34^

In this study, we considered the position of the first occurrence of medium risk (the lowest median risk), corresponding to the recommended frequency of QC tests. First, the S and O values were determined according to Section 2.3; Second, the S and O values were marked at the risk level corresponding to RM; Finally, the risk levels at different frequencies were plotted on the frequency (Y‐axis) and S (X‐axis) two‐dimensional map.

**FIGURE 2:**
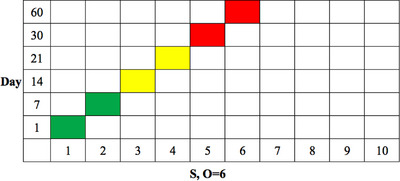
The RM for a QC item with six different frequencies.

With the S and O indices known for a given FM, the corresponding cell can be found from Figure 1, and its color indicates the risk level. For a given QC item, there are six FMs, which correspond to six different frequencies that share the same O index. The corresponding cells found in Figure 1 can be combined into one plot. The rank of index S is plotted along the X‐axis, and the frequency is plotted along the Y‐axis, as shown in Figure 2. As Figure 2 shows, when QC is performed at intervals of 30 days (D30) or 60 days (D60) the risk level is unacceptable (the red cells). If QC is performed at intervals less than 30 days (D30), the risk level is conditionally acceptable (the yellow cells). Therefore, the candidate frequencies for this QC item are D1–D21 (corresponding to the green and yellow cells in Figure 2). In this example, the suggested frequency is D14, which maintains an optimal balance between an acceptable risk level and minimal human effort.

### 2.5 Performance evaluation

To evaluate the performance of the proposed method, we measured the six QC items at both the new and old frequencies. We collected the evaluation dataset from the same Unity MR‐linac between January 2022 and March 2022. If the two frequencies for a given QC item were the same, the new frequency was consistent with the old one. When the two frequencies were different, we evaluated the effectiveness of the new frequency using the O and D indices. We defined an evaluation metric E = O/D, which characterizes the ability of the given QC frequency to detect machine failures.^24^ If the new frequency is lower than the old one, O increases and D decreases, increasing E, which indicates that more failures are detected earlier.

## 3 RESULTS

### 3.1 The FM indices

**FIGURE 3:**
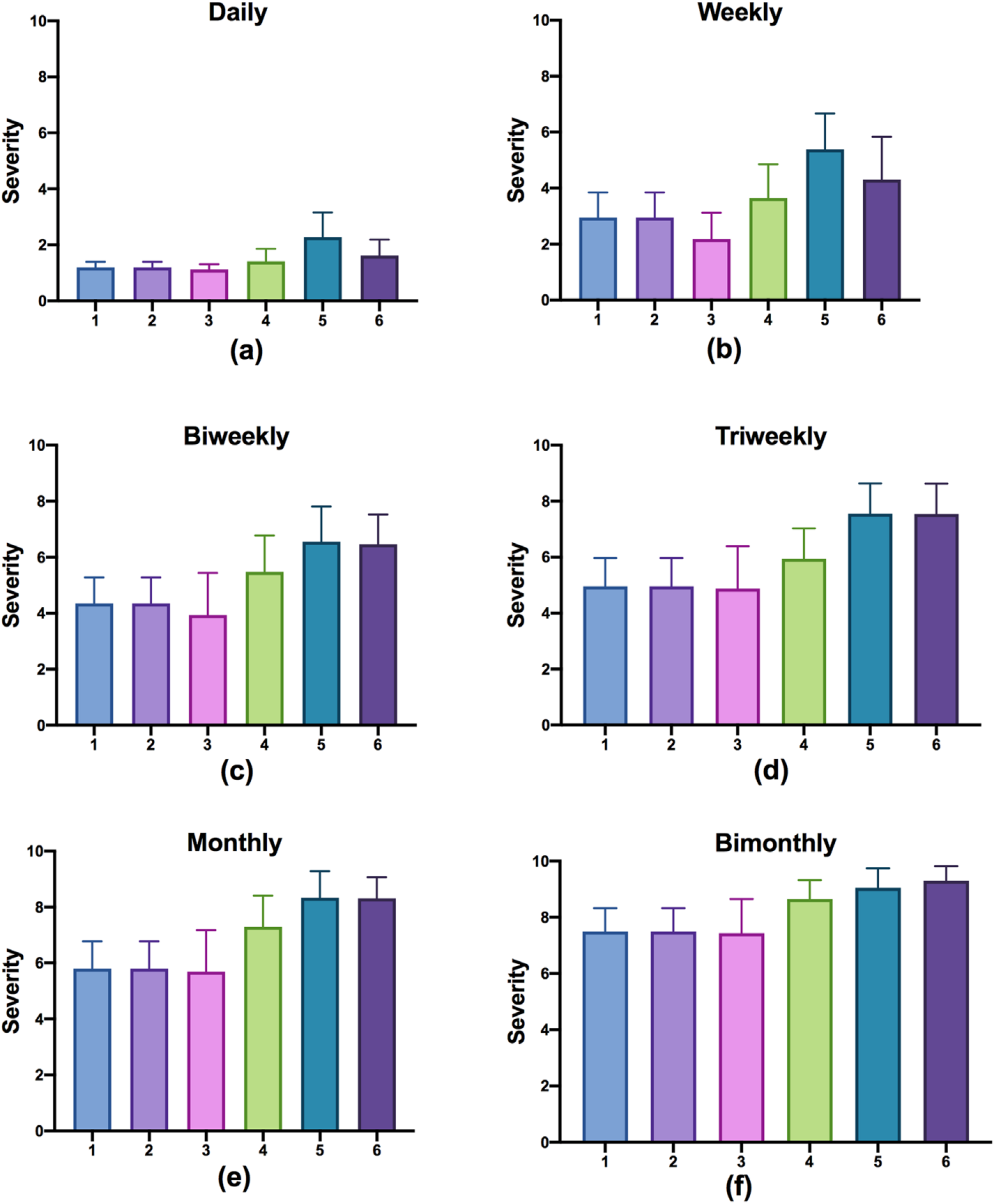
The S indexes for six QC items at each of six frequencies: (a) daily, (b) weekly, (c) biweekly, (d) triweekly, (e) monthly, and (f) bimonthly.

Figure 3 shows the S indices for all six QC items at each of the six frequencies. In each plot, the mean values of S are shown as colored bars, and the 95% confidence intervals are shown as error lines for all six QC items at a given frequency. This figure shows that S is smallest for the daily QC frequency. As the QC interval increases, S increases correspondingly. This result was consistent with the routine QC record.

Table 4 summarizes the S and O indices for all 36 FMs. The gantry angle (QC1) was adjusted more frequently, covering 0.278% of total daily operations, so the corresponding value of O was largest for it. Since the S and O indices represented the risk levels without QC inspections, the priorities of these QC items could be ranked accordingly.

**TABLE 4:** The indexes of S and O for all 36 FMs.

QC item	S						
No.	D1	D7	D14	D21	D30	D60	O*
1	1.200	2.950	4.350	4.950	5.800	7.500	6
2	1.200	2.950	4.350	4.950	5.800	7.500	2
3	1.125	2.188	3.938	4.875	5.688	7.438	2
4	1.412	3.647	5.471	5.941	7.294	8.647	2
5	2.278	5.389	6.556	7.556	8.333	9.056	2
6	1.615	4.308	6.462	7.538	8.308	9.308	2

* O is the same for the 6 FMs (D1, D7, D14, D21, D30, and D60) related to a QC item.

### 3.2 QC frequency

**FIGURE 4:**
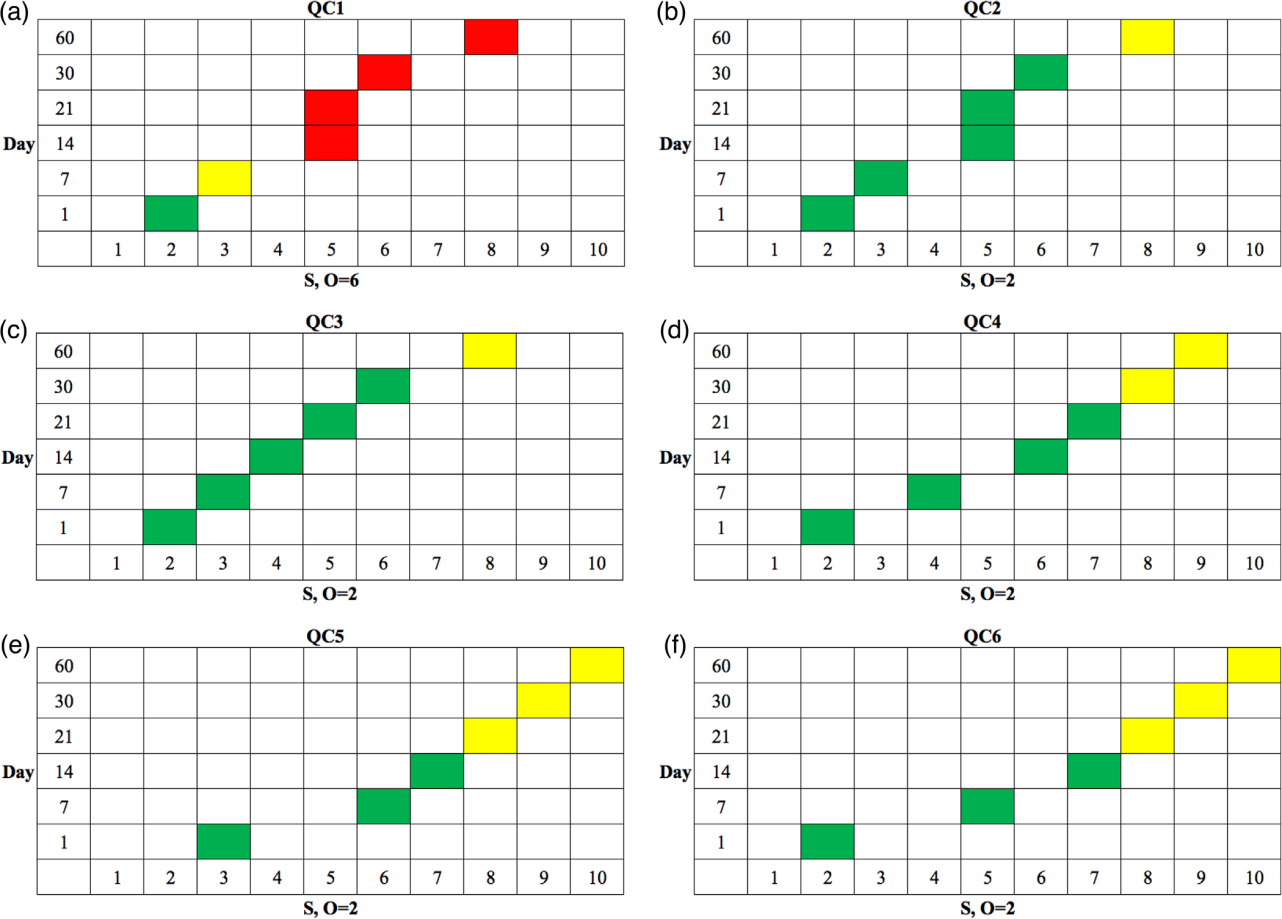
The RM plots for all six QC items at each of six frequencies: (a) QC1 with O = 6, (b) QC2 with O = 2, (c) QC3 with O = 2, (d) QC4 with O = 2, (e) QC5 with O = 2, and (f) QC6 with O = 2.

Figure 4 shows the RM plots for all six QC items at each of the six frequencies. The optimal QC frequency can be found from the earliest occurrence of a yellow cell (medium risk). These frequencies were D7 for QC1, D60 for QC2, D60 for QC3, D30 for QC4, D21 for QC5, and D21 for QC6. This indicated that QC1 should be tested more frequently, while QC2 and QC3 could be tested less frequently.

Table 5 compares the frequencies for all six QC items obtained with our method to the frequencies recommended by the vendor report. One new frequency (QC4) was the same as the vendor‐specified frequency, two new frequencies (QC2 and QC3 both bimonthly) were lower than the vendor‐specified frequencies, and three new frequencies (QC1 weekly, QC5 triweekly, and QC6 triweekly) were higher than the old ones.

**TABLE 5:** The comparison of frequencies calculated by the proposed method and recommended by the vendor report.

QC item No.	Proposed method	Vendor report
1	Weekly	Monthly
2	Bimonthly	Monthly
3	Bimonthly	Monthly
4	Monthly	Monthly
5	Triweekly	Monthly
6	Triweekly	Monthly

### 3.3 Performance evaluation

Table 6 shows the index E obtained using the new and old frequencies. The values of E for items QC1 and QC6 at the new frequencies were higher than those obtained at the old frequencies. The values of E for QC2, QC3, QC4, and QC5 were the same for both the new and old frequencies. In general, the values of E at the new frequencies were not less than those at the old frequencies. This meant that testing these QC items at the new frequencies improved detections of the risk of machine failure.

**TABLE 6:** The comparison of E resulted by the new and old frequencies.

	New frequency			Old frequency		
QC test	O	D	E	O	D	E
QC1	2	1	2.00	2	5	0.40
QC2	2	1	2.00	2	1	2.00
QC3	2	1	2.00	2	1	2.00
QC4	2	1	2.00	2	1	2.00
QC5	2	1	2.00	2	1	2.00
QC6	2	6	0.33	2	7	0.29

## 4 DISCUSSION

The suggested new frequency for QC1 is higher than the old one, which indicates a higher priority in preventing deviations of the gantry angle. The calculated frequencies for QC5 and QC6 are greater than the old ones, which indicate that higher priorities are required to maintain the isocenter accuracy. Although failures of QC5 and QC6 have not been found so far, if they were to occur, they would cause higher severity, so they still require attention. The calculated frequencies for QC2 and QC3 are lower than the old ones; thus, they would maintain a high level of machine performance while saving physicists’ efforts.

The performance evaluations of the new frequencies show that the values of E for QC1 and QC6 are largest compared with the values corresponding to the old frequencies. This indicates that the new frequencies are more effective in detecting potential errors of the Unity MR‐linac. Further, the new frequencies for QC2 and QC3 are lower than the old frequencies, but the value of E has not changed. This indicates that the new frequencies can save physicists’ efforts in performing linac QC. The new frequency for QC5 is higher than the old frequency, but again the corresponding value of E is unchanged. This is because the severity of QC5 is higher than that of the other items. O'Daniel^22^ proposed that the largest risk probability number (RPN, the multiplying O, S, and D) for daily QC can be used to determine the appropriate QC frequency. However, the frequency based on the RPN may not reflect the actual risk. A high‐RPN failure does not necessarily indicate a high risk for the process. The risk matrix is determined by S and O values according to a two‐dimensional look‐up table, which determines its risk level. The RPN is obtained by multiplying O, S and D, and its magnitude determines which step is given priority attention. Moreover, two failure modes with the same RPN value may not have the same risk level in the RM.

The frequencies for testing QC items recommended in the vendor report are based on the experiences of clinical experts, and it serves as the QC foundation for the MR‐linac system.^35^ Compared to a conventional linac,^1^ QC for the MR‐linac has two additions: MR tests and MRI‐to‐MV coordinate system checks.^30^ For QC1 (the gantry angle indicator), the tolerance in the vendor report is 0.3°, while that in our institute is 0.2°. The QC frequency recommended for this item by the vendor report is monthly, while that recommended by RM analysis is weekly; this demonstrates that a stricter tolerance results in a higher QC frequency. For items QC2 and QC3, the new QC frequencies are lower, reducing the time and effort spent on machine QC. This shows that risk management can validate the rationality of QC frequency.

In the proposed method, the QC frequency is solely determined by the indices S and O and not by the index D; this may overestimate the frequencies for some QC items. In clinical practice, the QC frequency is usually fixed (daily, monthly, and annual), regardless of changes in the status of the linac. Thus, an adaptive adjustment of the QC frequency is preferable, but its effectiveness must be validated. So far, we have demonstrated that the proposed method is applicable for regular mechanical QC tests. However, for imaging and dosimetric QC tests, an extension of the proposed method is needed, and this is currently under development.

Although we have tested the proposed method on a newly installed Unity MR‐linac, the method is not limited to the QC items employed in this study. It can easily be extended to other QC items (daily, monthly, or annual items) that are specifically designed for different radiotherapy machines. Therefore, the clinical feasibility and effectiveness of the proposed method can be evaluated for various linacs and for different treatment techniques. AAPM TG 100 report^24^ recommends performing appropriate tests to detect errors connected to the specific use of the linac, and this approach, while demanding, is in accordance with that advice.

## 5 CONCLUSION

In this study, we have demonstrated the use of RM analysis to determine the optimal frequencies for performing QC tests of a given treatment platform. This approach is able to identify optimal QC frequencies based on the regular QC schedule and service records. This application of RM analysis provides an effective way of evaluating the risk of various QC items for a radiotherapy machine. Thus, it can be used to produce an efficient QC scheme that maintains higher performance of a radiotherapy machine while simultaneously saving physicists’ efforts.

## AUTHOR CONTRIBUTIONS

Min Ma: study conception, design, data acquisition, wrote paper; Hui Yan, Minghui Li, Yuan Tian, Ke Zhang, and Kuo Men: drafted the manuscript; Jianrong Dai: drafted the manuscript and supervised the study; and all authors revised it critically for important intellectual content. All have given final approval.

## CONFLICT OF INTEREST STATEMENT

The authors declare no conflicts of interest.

## Data Availability

Research data are not available at this time.
